# Xanthohumol, a Prenylated Chalcone Derived from Hops, Inhibits Growth and Metastasis of Melanoma Cells

**DOI:** 10.3390/cancers13030511

**Published:** 2021-01-29

**Authors:** Tatjana Seitz, Christina Hackl, Kim Freese, Peter Dietrich, Abdo Mahli, Reinhard Manfred Thasler, Wolfgang Erwin Thasler, Sven Arke Lang, Anja Katrin Bosserhoff, Claus Hellerbrand

**Affiliations:** 1 Institute of Biochemistry (Emil-Fischer-Zentrum), Friedrich-Alexander University Erlangen-Nürnberg, D-91054 Erlangen, Germany; tatjana.seitz@fau.de (T.S.); kim.freese@fau.de (K.F.); peter.dietrich@fau.de (P.D.); abdo.mahli@fau.de (A.M.); anja.bosserhoff@fau.de (A.K.B.); 2 Department of Internal Medicine I, University Hospital Regensburg, D-93053 Regensburg, Germany; 3 Department of Surgery, University Hospital Regensburg, D-93053 Regensburg, Germany; christina.hackl@ukr.de; 4 Medical Clinic 1, Department of Medicine, University Hospital Erlangen, Friedrich-Alexander-University, D-91054 Erlangen, Germany; 5 Stiftung HTCR, BioPark III, Am BioPark 13, D-93053 Regensburg, Germany; reinhard.thasler@htcr.de; 6 Hepacult GmbH, D-82152 Martinsried, Germany; wolfgang.thasler@hepacult.de; 7 Department of Surgery and Transplantation, University Hospital RWTH Aachen, D-52074 Aachen, Germany; svlang@ukaachen.de; 8 Comprehensive Cancer Center (CCC) Erlangen-EMN, D-91054 Erlangen, Germany

**Keywords:** xanthohumol, melanoma, metastasis

## Abstract

**Simple Summary:**

Melanoma is an aggressively growing form of skin cancer. It has a high metastatic potential, and the liver is one of the most common sites for visceral metastasis. Patients with hepatic metastases have a very poor prognosis, and effective forms of treatment are urgently needed. Xanthohumol is a natural compound of the hop plant with a wide range of beneficial health effects. In the present study, we show in cell culture experiments with human melanoma cells that xanthohumol inhibits several pro-tumorigenic mechanisms that are critical for melanoma metastasis. Furthermore, we analyzed the effect of xanthohumol application in a mouse model of melanoma metastasis and found that xanthohumol significantly inhibited the growth of melanoma metastases in the liver. Together with previous studies indicating that xanthohumol application is well tolerated, our present findings point to this natural compound as a promising novel treatment of melanoma patients with liver metastases.

**Abstract:**

Melanoma is one of the most aggressive and lethal cancers worldwide. Despite recent progress in melanoma therapy, the prognosis for metastasized melanoma continues to be poor. Xanthohumol (XN), a prenylated chalcone derived from hop cones, is known to possess a broad spectrum of chemopreventive and anticancer activities. However, few studies have analyzed functional XN effects on melanoma cells and there have been no previous in vivo studies of its effects on metastasis. The aim of this study was to investigate the impact of XN on the tumorigenic and liver metastatic activity of melanoma cells. XN exhibited dose-dependent cytotoxic effects on human melanoma cell lines (Mel Ju; Mel Im) in vitro. Functional analysis in the subtoxic dose-range revealed that XN dose-dependently inhibited proliferation, colony formation, and migratory activity of melanoma cells. Subtoxic XN doses also induced markers of endoplasmic reticulum stress but inhibited the phosphorylation of the protumorigenic c-Jun N-terminal kinases (JNK). Furthermore, XN effects on hepatic metastasis were analyzed in a syngeneic murine model (splenic injection of murine B16 melanoma cells in C57/BL6 mice). Here, XN significantly reduced the formation of hepatic metastasis. Metastases formed in the liver of XN-treated mice revealed significantly larger areas of central necrosis and lower Ki67 expression scores compared to that of control mice. In conclusion, XN inhibits tumorigenicity of melanoma cells in vitro and significantly reduced hepatic metastasis of melanoma cells in mice. These data, in conjunction with an excellent safety profile that has been confirmed in previous studies, indicate XN as a promising novel agent for the treatment of hepatic (melanoma) metastasis.

## 1. Introduction

Melanoma is the tumor entity with the fastest rising incidence rates [1]. It accounts for the vast majority of skin cancer-related deaths [2], which are caused by its high metastatic potential. Although the majority of melanoma patients can undergo initially curative local resection of the primary tumor, around 30% of patients will recur with metastatic disease [3].

Melanoma has the propensity to metastasize to almost any organ, with the liver being one of the most common sites for visceral metastasis [4]. Hepatic metastasis of malignant melanoma has a very poor prognosis, with a median overall survival of 4–12 months in patients not undergoing liver metastasectomy and 14–41 months following liver resection [5]. Therefore, effective management of (hepatic) metastasis represents a critical clinical challenge.

Development of an immune checkpoint blockade and highly effective targeted therapies (BRAF and MEK inhibition) extended the treatment options for patients with distant metastases [6]. However, the prognosis for metastasized melanoma continues to be poor, underscoring the need for novel therapies [7].

Considerable interest is growing in the anti-tumorigenic effects of xanthohumol (3′-[3,3-dimethyl allyl]-2′,4′,4-trihydroxy-6′-methoxychalcone), the principal prenylated flavonoid of the hop plant (*Humulus lupulus* L.). Xanthohumol (XN) has been claimed to exert a broad range of biological activities, including anti-angiogenic [8,9], anti-oxidative [10,11], and chemopreventive properties [10]. In particular, XN attracted attention for its efficacy against several types of cancer, e.g., hepatocellular carcinoma [12,13,14], prostate cancer [15,16,17,18], and breast cancer [16,19,20,21]. As an antitumor agent, XN has been widely studied for its anti-inflammatory effects [14,22].

Although XN is known as a broad-spectrum chemotherapeutic agent, only one study has analyzed anti-tumorigenic effects of XN on murine melanoma cells in vitro [23] and no in vivo studies of XN effects on metastasis of any tumor entity exist.

Therefore, the aim of this study was to analyze the functional effects of XN on melanoma cells in vitro and in a syngeneic in vivo model of hepatic metastasis. 

## 2. Results

### 2.1. Effects of Xanthohumol on Viability of Melanoma Cells

**Figure 1:**
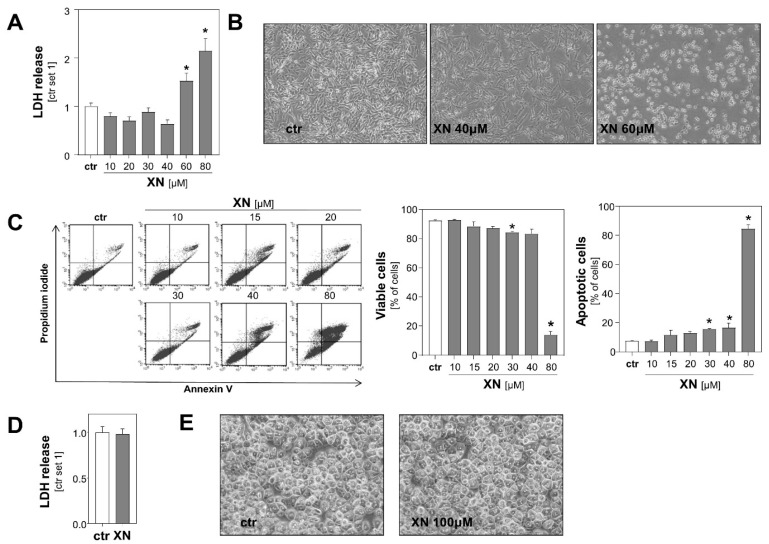
Effect of xanthohumol (XN) on viability of melanoma cells and primary human hepatocytes. (**A**) Quantification of lactate dehydrogenase (LDH) release into the supernatant and (**B**) representative microscopic images of human melanoma cells (Mel Ju) treated with different doses of XN for 48 h. (**C**) Flow cytometric analysis of Annexin V-FITC and propidium iodide (PI)-stained Mel Ju cells following treatment with indicated XN concentrations for 24 h. Proportion of viable cells (middle panel), apoptotic cells (right panel), and representative dot plots (left panel) are shown. (**D**) Quantification of LDH release into the supernatant of primary human hepatocytes following treatment with 100 μM XN for 48 h and (**E**) representative microscopic images. (*: *p* < 0.05 compared with ctr.).

Initially, we determined the range of toxicity of xanthohumol (XN) in the two human melanoma cell lines Mel Ju and Mel Im. Analysis of lactate dehydrogenase (LDH) levels in the supernatant and microscopic analysis revealed that XN exhibited dose-dependent cytotoxic effects on both melanoma cell lines beginning in the dose range of 40–60 µM (Figure 1A,B, Figure S1). Consistent with this, Annexin V-FITC flow cytometric analysis showed that viability of cells was only slightly impaired following incubation with XN up to 40 μM for 24 h (Figure 1C). Incubation with 40 μM XN resulted in only a slight increase of apoptotic cells (Annexin V positive cells) (Figure 1C), and no significant propidium iodide (PI) incorporation was observed at any XN concentration up to 30 µM (data not shown). In contrast and in line with previous reports [14], XN doses up to 100 µM did not cause any signs of toxicity in primary human hepatocytes (Figure 1D,E). These data indicated the vulnerability of malignant melanoma cells to XN treatment compared to non-malignant liver cells.

### 2.2. Xanthohumol Effects on Growth and Migration of Melanoma Cells In Vitro

**Figure 2:**
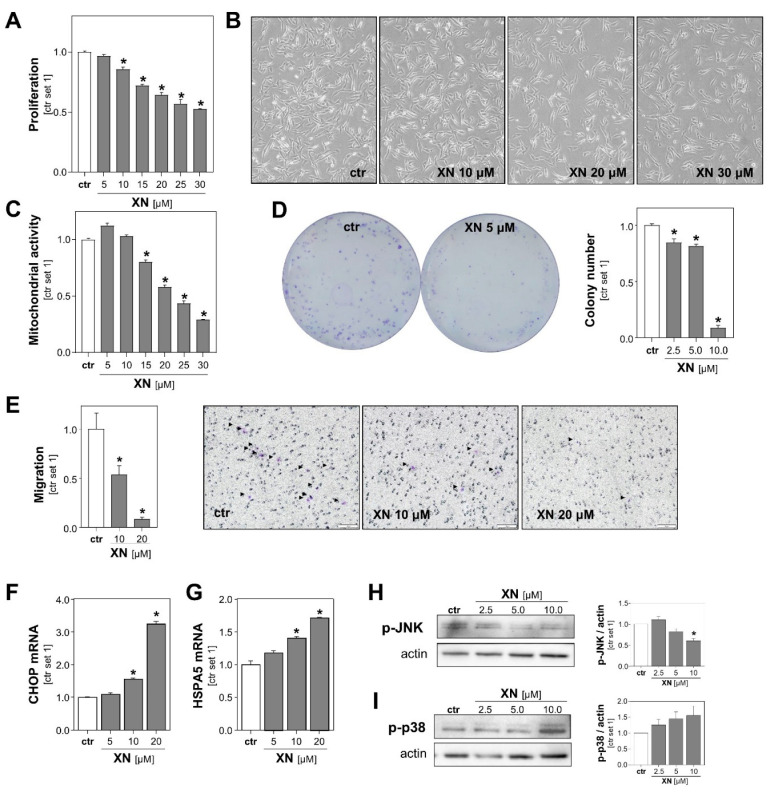
Functional effects of xanthohumol (XN) on melanoma cells in vitro. (**A**) Proliferation, (**B**) representative microscopic images, and (**C**) mitochondrial activity of Mel Ju cells following treatment with subtoxic doses of XN. (**D**) Quantification of colony number (right panel) and representative images (left panel) in anchorage-dependent clonogenic assays with Mel Ju cells treated without (ctr) or with XN. (**E**) Migratory activity of Mel Ju cells following 4 h treatment with indicated doses of XN (left panel) and representative images of Boyden chamber filters (right panel). Arrowheads indicate migrated cells. (**F**) CHOP and (**G**) HSPA5 mRNA expression levels in Mel Ju cells treated without (ctr) or with XN. Western blot analysis of (**H**) phosphorylated JNK1/2 and (**I**) p38 in XN-treated and control (ctr) Mel Ju cells (representative images (left panels) and densitometric quantification (right panels)). (*: *p* < 0.05 compared with ctr.).

To further characterize the effect of XN on melanoma cells, we performed functional in vitro assays with both melanoma cell lines treated with non-toxic XN concentrations (up to 30 µM). A fluorescence-based DNA quantification assay revealed that proliferation of Mel Ju and Mel Im cells declined progressively with increasing concentrations of XN (Figure 2A, Figure S2A). Microscopic imaging also showed a dose-dependent decrease in melanoma cell number following incubation with XN for 24 h (Figure 2B, Figure S2B). In line with this, XN treatment led to a dose-dependent reduction in mitochondrial activity of melanoma cells (Figure 2C, Figure S2C left panel), which was determined by analysis of the reduction of XTT (sodium 3′-[1-(phenylaminocarbonyl)-3,4-tetrazolium]-bis (4-methoxy6-nitro) benzene sulfonic acid hydrate). Previous studies have shown in other cancer cell types, such as colon and breast cancer cells [24,25], that XN exhibits parts of its anti-tumorigenic effects via impairment of mitochondria. Here, we analyzed the effect of XN on mitochondrial activity in primary human hepatocytes (PHHs) by applying the XTT-assay. We found that mitochondrial activity was not reduced but even slightly increased in XN-treated compared with control PHHs (Figure S2C right panel).

Next, we assessed the impact of XN on melanoma cells in clonogenicity assays. This assay analyzes stem cell properties, anchorage-dependent colony formation, and growth of cancer cells. We found that XN significantly reduced the colony number as well as the colony size of both melanoma cell lines. Colony formation was almost completely inhibited with XN concentrations as low as 10 μM (Figure 2D, Figure S2D).

Next, we analyzed XN effects on the migration capability of melanoma cells using a Boyden chamber assay. Migration was analyzed after only 4 h of incubation to exclude anti-proliferative effects of XN. We observed that the migratory activity of XN-treated melanoma cells was significantly reduced compared to that of control cells (Figure 2E, Figure S2E). Together, these data showed that in addition to its cytotoxic effects, even subtoxic XN concentrations inhibit in vitro pathological functions of melanoma cells that are critical for tumor growth and metastasis.

To identify the mechanism through which XN was exhibiting its functional effects on melanoma cells, we analyzed its effects on the expression of markers of endoplasmic reticulum (ER) stress. Previous studies have shown that XN induced ER-stress in leukemia and breast cancer cells [26,27], and a recent study by Zhang et al. found that ER-stress mediated XN-induced cell death in a murine melanoma cell line [23]. Interestingly, we also observed that in the subtoxic range: XN dose-dependently induced the expression of the ER stress markers CCAAT-enhancer-binding protein homologous protein (CHOP) and heat shock protein family A (Hsp70) member 5 (HSPA5; also known as binding immunoglobulin protein (BiP)) in both human melanoma cell lines (Figure 2F,G, Figure S2F,G). Furthermore, we analyzed the effects of XN on the phosphorylation of c-Jun N-terminal kinases (JNK) and found that XN inhibited the JNK-phosphorylation in the subtoxic range (Figure 2H, Figure S2H). Importantly, this mitogen-activated protein kinase (MAPK) has been shown to be a critical promotor of different aspects of the tumorigenicity of melanoma cells [28]. In contrast, phosphorylation of p38 MAPK was slightly induced following treatment with XN (Figure 2I, Figure S2I). Interestingly, a study by Mi et al. indicated that XN-induced cell death of leukemia cells is mediated by the p38 MAPK signaling pathway [29]. The role of p38 in apoptosis of melanoma cells is controversial, and opposing pro- and anti-tumorigenic effects have been described [30].

### 2.3. Effect of Xanthohumol on Hepatic Metastasis of Melanoma Cells In Vivo

**Figure 3:**
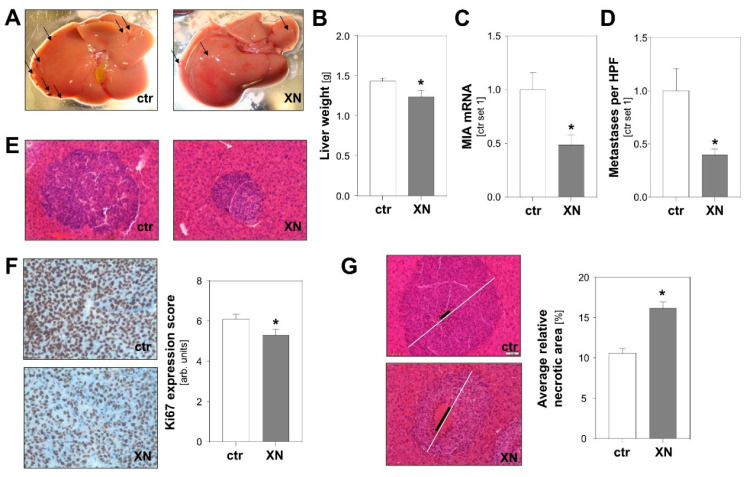
Effect of xanthohumol (XN) on hepatic metastasis of melanoma cells in a syngeneic in vivo model. Mice were implanted with either XN-liberating pellets (10 mg/kg body weight; *n* = 7 mice) or control pellets (ctr.; *n* = 10 mice). (**A**) Representative macroscopic liver images (arrows indicate exemplary metastases on liver surface). (**B**) Liver weight and (**C**) hepatic MIA mRNA expression as markers of hepatic tumor burden. (**D**) Average number of metastases per high-power field (HPF) in XN-treated and control (ctr) mice. (**E**) Representative images of HE stained hepatic tissues (20-fold magnification). (**F**) Immunohistochemical Ki67 staining of hepatic metastases (left panel) and analysis of Ki67 expression (right panel). (**G**) Representative images of hepatic metastases with similar size in the two mice groups. White bar indicates diameter of the metastasis and black bar the diameter of central necrosis (left panel); quantification of relative necrotic area (right panel). (*: *p* < 0.05 compared with control).

To test the effect of XN on tumor metastasis in vivo, we employed an established syngeneic murine model of hepatic metastasis [31,32]. Murine B16-F10 melanoma cells were injected into the spleen of syngeneic Bl/6N mice with and without XN treatment. XN was applied via intraperitoneally implanted pellets releasing XN continuously over time with a daily dose of 10 mg/kg body weight [33]. Control mice received vehicle-containing placebo pellets. Macroscopic analysis showed less metastases on the liver surface of XN-treated mice (Figure 3A). Similar to what was observed in previous studies [31], metastases appeared in part as whitish, a sign of depigmentation. Interestingly, a recent study found that loss of melanin pigmentation enhances the ability of melanoma cells to spread in vivo [34]. Here, we observed that liver weight was significantly lower in XN-treated mice reflecting reduced hepatic tumor burden (Figure 3B). Tumor load was also quantified by analysis of melanoma cell-specific MIA expression in hepatic tissues. This gene is specifically expressed in melanoma cells but not in liver tissue [35,36]. Quantitative RT-PCR analysis revealed significantly lower MIA mRNA levels in livers of XN-treated animals reflecting reduced hepatic tumor load (Figure 3C). In line with this, histological analysis showed fewer and smaller metastases in XN-treated mice (Figure 3D,E). Immunohistological analysis revealed significantly reduced Ki67 staining in metastases formed in XN-treated mice, indicative of reduced mitotic activity of melanoma cells in these tumors (Figure 3F). Furthermore, XN treatment resulted in significantly larger areas of central necrosis in hepatic metastases (Figure 3G). In summary, these data demonstrate that XN significantly reduced hepatic metastasis of melanoma cells in vivo.

## 3. Discussion

In many cancer entities, including melanoma, hepatic metastasis is the critical factor determining tumor-associated mortality [37]. Despite improvements in comprehensive therapies for malignant melanoma, treatment of hepatic metastasis is still challenging due to limited response rates and rapid onset of resistance [6]. Furthermore, targeted therapies are often associated with serious adverse effects [38].

The low toxicity and the excellent safety profile of xanthohumol (XN) has been demonstrated in several studies (as reviewed in [39,40]). A number of studies provide substantial evidence that this hop-derived prenylated chalcone exhibits antitumor activity against a wide array of cancer cells, such as hepatocellular carcinoma [12,13,14], breast cancer [16,19,20,21], and prostate cancer [15,16,17,18]. However, little was known about the functional effects of XN on melanoma cells, and to the best of our knowledge, no studies of in vivo effects of XN on metastasis of any tumor entity existed previously. Here, we show that XN exhibits in a dose-dependent manner the cytotoxic effects on human melanoma cell lines Mel Im and Mel Ju with effective potencies in the micromolar range (40–60 µM). These cytotoxic concentrations were comparable to that observed in other cancer cells; Dorn et al. observed induction of cell death in HCC cells upon stimulation with 25 µM XN [14] and Cho et al. reported half-maximal inhibitory concentrations of 10 µM for breast cancer cells [41]. In addition to cytotoxic effects, we also analyzed functional effects of XN on melanoma cells. Previous studies illustrated the anti-proliferative effects of XN on various cancer cell lines, including HCC cells [14], medullary thyroid cancer cells [42], and colon carcinoma cells [43]. Nonetheless, the current study shows, for the first time, that incubation with XN in the subtoxic range dose-dependently inhibits proliferation of human melanoma cells.

Besides proliferation, the migratory capability is one of the critical factors for cancer progression [44]. Here, we show that XN treatment inhibits migration of melanoma cells in concentrations as low as 10 µM. Notably, assay conditions were chosen to exclude cytotoxic or anti-proliferative effects of XN. Consistent with our data, previous studies have demonstrated the inhibitory effects of XN on migration of other cancer cells, e.g., HCC cells [14] and prostate cancer cells [17].

The liver is a common site for metastatic spread of malignant melanoma [4]. Thus, the in vitro results prompted us to investigate the effect of XN in an experimental in vivo model of hepatic metastasis. Here, for the first time, our study provides evidence for the anti-metastatic potential of XN in vivo. Adjustment of dose or dosing regimen may further increase the therapeutic efficacy. Together, these findings strongly reinforce XN as a promising agent for the treatment of hepatic melanoma metastases. Future preclinical studies need to address the potential for the treatment of melanoma metastasis to extrahepatic sites.

For a mechanistic study, we analyzed the extent of proliferating cells in hepatic metastases of XN-treated mice and controls by Ki67 staining. Remarkably, metastases of the XN group exhibited significantly lower Ki67 expression scores. Consistent with our in vitro data, this indicates that the inhibitory effect of XN on hepatic metastasis is, at least in part, mediated by anti-proliferative properties. Furthermore, it has been shown that ER-stress mediates XN-induced cell death [23,26,27], and interestingly, we found that in the subtoxic range, XN dose-dependently induced the expression of ER-stress markers CHOP and HSPA5. Even if ER-stress induced by lower XN doses does not yet cause (programmed) cell death, it may make melanoma cells more vulnerable. This hypothesis nicely fits to our observation of larger areas of necrosis within the metastases formed in the liver of XN-treated mice. Within the center of the metastases, hypoxia and nutrient starvation cause additional ER-stress, and thus, XN treatment may tip the balance towards melanoma cell death.

Nevertheless, it is important to take into account that further XN-mediated anti-tumorigenic mechanisms could contribute to the decreased tumor burden in this model. The anti-migratory activity of XN on melanoma cells, which has been demonstrated in vitro, might also be one of the mechanisms underlying reduced hepatic metastasis in XN-treated mice. Furthermore, the larger areas of central necrosis in metastases of XN treated mice might also be attributed to an inhibition of angiogenesis, which is crucial for the growth of metastases [45]. Indeed, previous studies elucidated the anti-angiogenic potential of XN in pancreatic cancer [8] and in hematologic malignancies [46]. Together, it has been shown that XN inhibits several pro-tumorigenic mechanisms and pathways in melanoma as well as cancers of other origin. Likely, several anti-tumorigenic XN effects together played a role in this in vivo model of hepatic melanoma metastasis. Further studies are required to elucidate whether the mode of action also varies during the course of metastasis or in metastasis to organs other than the liver. This could also be important information for the potential combined application with other more-targeted forms of therapy.

Insights in the safety profile are essential to using XN for therapeutic application. Previous studies have demonstrated the safety of even long term application of daily XN doses as high as 1.000 mg/kg body weight [47]. More recently, the safety of XN was also shown in a single-dose pharmacokinetic study performed on healthy volunteers [48] and an escalating dose study in menopausal women [49]. These studies indicate that XN has an excellent safety profile.

Together with our present findings, XN appears as a promising agent for the treatment of hepatic (melanoma) metastases. Since XN is known for its broad-spectrum activity against a wide array of cancers, it might also be efficient against (hepatic) metastasis from other tumor entities. This issue should be addressed in future studies. Moreover, the molecular mechanisms by which XN reduces hepatic metastasis in mice need to be further characterized. Thus, future in vitro and in vivo studies will expand the understanding of the anti-metastatic potential of XN and will guide future research on the use of this natural compound against metastatic disease.

## 4. Materials and Methods 

### 4.1. Cells and Cell Culture

The murine melanoma cell line B16-F10 was obtained from American Type Culture Collection (ATCC; CRL-6475™). The human melanoma cell lines Mel Ju and Mel Im were derived from melanoma metastases [50]. Cells were cultured as described [32]. Primary human hepatocytes (PHH) were isolated and cultured as described [51,52].

Cells were treated with xanthohumol (NIC Nookandeh Institute GmbH, Homburg/Saar, Germany) with different concentrations and time periods as indicated. The vehicle ethanol alone served as control.

### 4.2. In Vivo Model of Hepatic Metastasis

A previously described mouse model of hepatic metastasis was used [31,32]. Murine B16-F10 melanoma cells (2 × 10^5^ in 5 μL) were injected into the spleen of syngeneic Bl/6N mice (control, *n* = 10; XN, *n* = 7, age: 10 weeks, Charles River Laboratories, Sulzfeld, Germany) using a 10 μl Hamilton syringe (Novodirect GmbH, Kehl/Rhein, Germany). XN was applied via intraperitoneally implanted pellets (Innovative Research of America, Sarasota, FL, USA) releasing XN continuously over time with a daily dose of 10 mg/kg body weight. Control mice received control pellets. After 11 days, mice were sacrificed and the livers were processed for further analyses.

### 4.3. In Vitro Analysis of Cell Death and Cell Proliferation

LDH release into the supernatant was determined using the Cytotoxicity Detection KitPLUS (Roche Applied Sciences, Indianapolis, IN, USA).

For analysis of apoptosis, cells were stained with FITC-conjugated Annexin V and propidium iodide using the Annexin V-FITC Detection Kit (PromoKine, PromoCell GmbH, Heidelberg, Germany) according to the manufacturer’s instructions as described [51].

Mitochondrial activity of cells was determined using a colorimetric XTT assay (Roche Diagnostics, Mannheim, Germany) according to the manufacturer’s protocol, and was described previously [51].

Cell proliferation was analyzed the using a CyQUANT NF Cell Proliferation Assay Kit (Molecular Probes, Eugene, OR, USA) following the manufacturer’s description as described [51].

### 4.4. Analysis of Cell Migration

Migratory activity of melanoma cells following treatment with XN for 4 h was quantified using a Boyden chamber assay as described [51], with DMEM supplemented with 20% FCS attached to the bottom chamber. Further, cell migration was assessed using the Cultrex 96 Well Cell Migration Assay (Trevigen, Gaithersburg, MD, USA) according to the manufacturer’s protocol as described [14].

### 4.5. Clonogenic Assay

Clonogenic assays were used to analyze stem cell properties, anchorage-dependent colony formation, and growth of cancer cells as described previously [51].

### 4.6. Histological Quantification of Hepatic Metastasis and Tumor Necrosis

Standard 4 μm sections of formalin-fixed and paraffin-embedded tissue blocks were hematoxylin and eosin (HE) stained to determine the average number of metastases per high-power field (HPF) and to compare the level of hepatic metastasis and associated necrosis between XN-treated and control animals. HE-stained sections were examined under an Olympus CKX41 microscope with the ALTRA 20 Soft Imaging System and CellA software version 2.6 (Olympus Soft Imaging Solutions GmbH, Münster, Germany).

To determine the average number of metastases per HPF, metastases were counted in five HPFs per section in two tissue sections per mouse (cut at 1200 µm intervals).

For quantification of relative necrotic area, 0–3 metastases showing necrosis (depending on the existence and size of metastases with necrosis) in four tissue sections per mouse (cut at 400 µm intervals) were photographed at 10× magnification. The area of metastasis and corresponding necrosis was then determined using ImageJ software (National Institutes of Health, Bethesda, MD, USA) [53]. Finally, the average relative necrotic area (%) of 4–12 metastases per mouse was calculated.

### 4.7. Immunohistochemical Analysis of Ki67

Immunohistochemical staining for Ki67 was performed using anti-Ki67 antibody (ab15580 from Abcam, Cambridge, UK) and standard protocols as described [51]. Ki67 expression score based on number of positively stained cells and staining intensity [54] was determined in five metastases per mouse in a blinded manner.

### 4.8. Analysis of mRNA Expression

Total RNA was extracted with a Maxwell^®^ 16 LEV simplyRNA Tissue Kit (Promega, Madison, WI, USA) using a Maxwell 16 Instrument (Promega, Madison, WI, USA) following the manual’s instructions. Reverse transcription and quantitative real-time polymerase chain reaction using a LightCycler 480 System (Roche Diagnostics, Mannheim, Germany) were performed as described [51]. The following sets of primers were used: 18S (forward: 5′-AAA CGG CTA CCA CAT CCA AG-3′, reverse: 5′-CCT CCA ATG GAT CCT CGT TA-3′), β-actin (forward: 5′-CTA CGT CGC CCT GGA CTT CGA GC-3′, reverse: 5′-GAT GGA GCC GCC GAT CCA CAC GG-3′), CHOP (forward: 5′-GTT AAA GAT GAG CGG GTG GCA G-3′, reverse: 5′-CAC TTC CTT CTT GAA CAC TCT CTC C-3′), HSPA5 (forward: 5′- CGT GGA ATG ACC CGT CTG TG-3′, reverse: 5′-GCC AGC AAT AGT TCC AGC GTC-3′), and MIA (forward: 5‘-CCA AGC TGG CTG ACT GGA AG-3′, reverse: 5′- GCC AGG TCT CCA TAG TAA CC-3′). Amplification of cDNA derived from 18S rRNA or β-actin was used for normalization of data.

### 4.9. Western Blot Analysis

Protein extraction, Western blotting, and densitometric quantification were performed as described [51]. The following primary antibodies were used: rabbit anti-phospho-JNK (#9251, 1:1000; Cell Signaling Technology), rabbit anti-phospho-p38 (#9215, 1:1000; Cell Signaling Technology), and mouse anti-actin (MAB1501, 1:10,000; Merck Millipore, Billerica, MA, USA). Mouse anti-rabbit (sc-2357; 1:10,000; Santa Cruz Biotechnology) and horse anti-mouse (#7076, 1:3000; Cell Signaling Technology) were used as secondary antibodies. Original, uncropped blots are shown in Figure S3.

### 4.10. Statistical Analysis

Statistical analysis was carried out using GraphPad Prism Software version 8.4.3 (GraphPad Software, San Diego, CA, USA). Data are shown as the mean ± standard error of the mean (SEM). Data sets were compared with analysis of two-tailed unpaired Student’s t-test. In vitro experiments were performed in biological triplicates, and the analysis of the presented data was performed in at least triplicates. A *p*-value < 0.05 was considered statistically significant.

## 5. Conclusions

In this study, we newly showed that xanthohumol (XN) inhibits tumorigenicity of melanoma cells in vitro and in vivo. In particular, we demonstrated in in vitro experiments that XN inhibits the proliferation and migratory ability of melanoma cells even in subtoxic concentrations. Most importantly, we provide the first experimental evidence that the prenylated chalcone XN significantly reduces hepatic metastasis of melanoma cells in mice. We propose that the antiproliferative effects of XN on melanoma cells play a key role in the mechanism of action. Our present findings, in conjunction with an excellent safety profile, point to XN as a promising agent for the treatment of hepatic (melanoma) metastases.

## Data Availability

Data is contained within the Results and Supplementary Material.

## Supplementary Materials

The following are available online at https://www.mdpi.com/2072-6694/13/3/511/s1. Figure S1: Effect of xanthohumol (XN) on viability of melanoma cells, Figure S2: Functional effects of xanthohumol (XN) on melanoma cells in vitro, Figure S3: Original, uncropped blots.
