# Supplementary material for: Xanthohumol, a Prenylated Chalcone Derived from Hops, Inhibits Growth and Metastasis of Melanoma Cells
Source: Cancers (Basel). 2021 Jan 29;13(3):511. doi: 10.3390/cancers13030511 (PMC7866261; doi:10.3390/cancers13030511)

SFigure 1

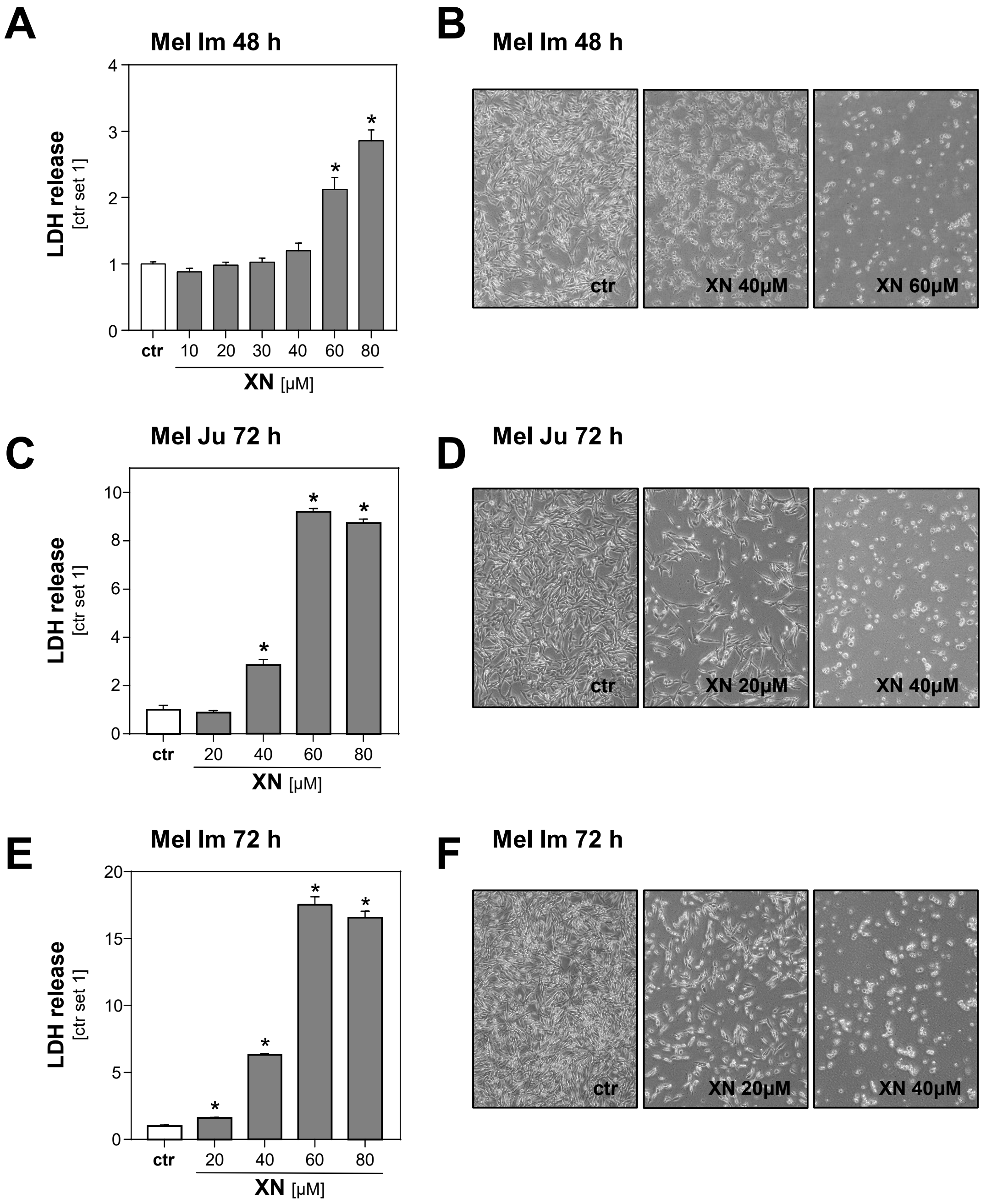

SFigure 1. Effect of Xanthohumol (XN) on viability of melanoma cells. Quantification of lactate dehydrogenase (LDH) release into the supernatant of (**A**) Mel Im cells treated with different doses of XN for 48 h, (**C**) Mel Ju cells treated with XN for 72h and (**E**) Mel Im cells treated with XN for 72 h. (**B, D, F**) Representative microscopy images corresponding to graphs shown in A, C, E. (*: $p < 0.05$).

SFigure 2

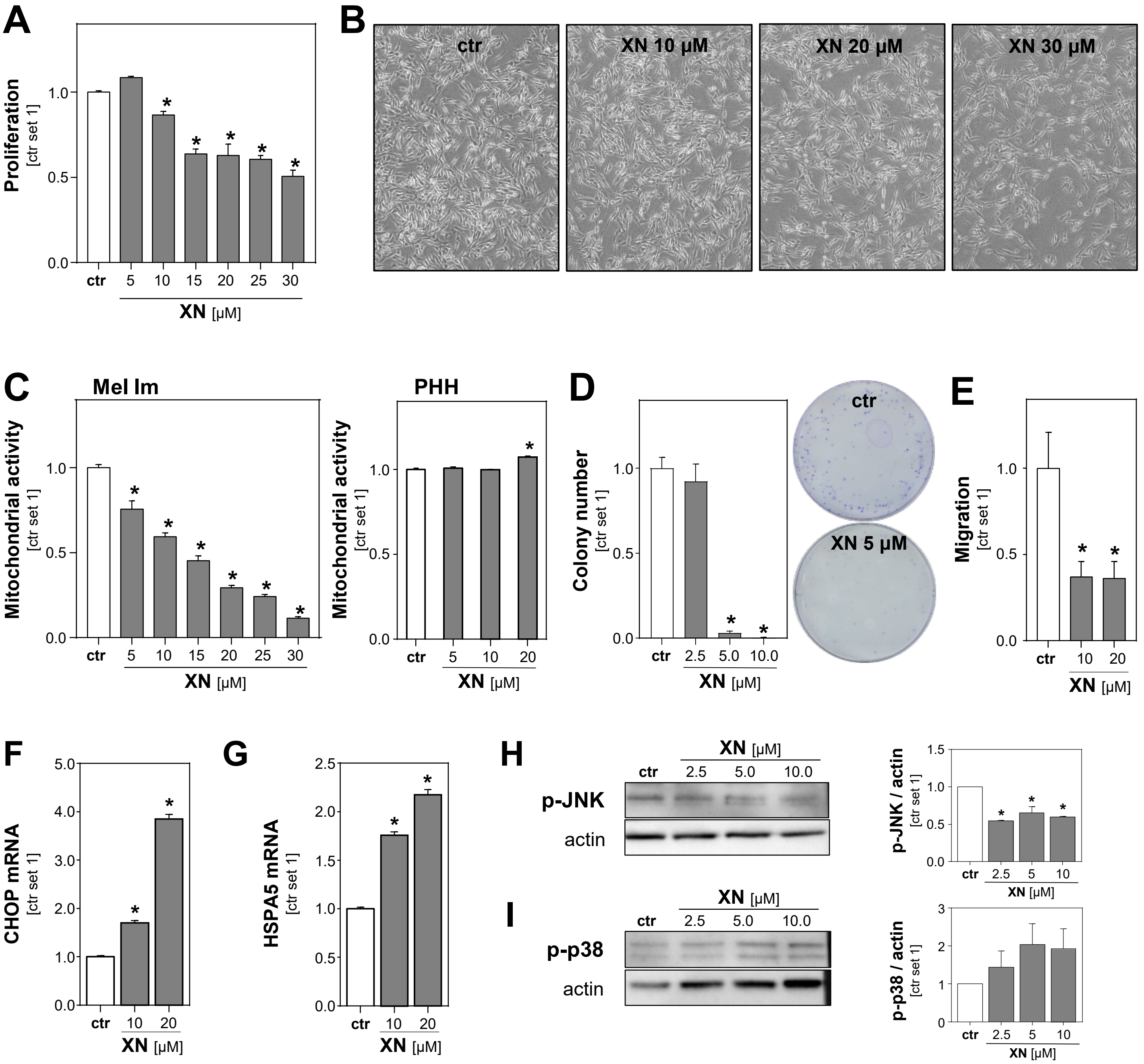

SFigure 2. Functional effects of Xanthohumol (XN) on melanoma cells *in vitro*. (**A**) Proliferation, (**B**) representative microscopy images and (**C**) mitochondrial activity of Mel Im cells (left panel) and primary human hepatocytes (PHH, right panel) following treatment with subtoxic doses of XN. (**D**) Quantification of colony number (left panel) and representative images (right panel) in anchorage-dependent clonogenic assays with Mel Im cells treated without (ctr) or with XN. (**E**) Migratory activity of Mel Im cells following 4 h treatment with indicated doses of XN. (**F**) CHOP and (**G**) HSPA5 mRNA expression levels in Mel Im cells treated without (ctr) or with XN. (**H**) Western Blot analysis of phosphorylated JNK1/2 in XN treated and control (ctr) Mel Im cells (representative images and densitometric quantification). (**I**) Western Blot analysis of phosphorylated p38 in XN treated and control (ctr) Mel Im cells (representative images and densitometric quantification). (*: $p < 0.05$).

SFigure 3

Original, uncropped blots of Fig.2H

pJNK

actin

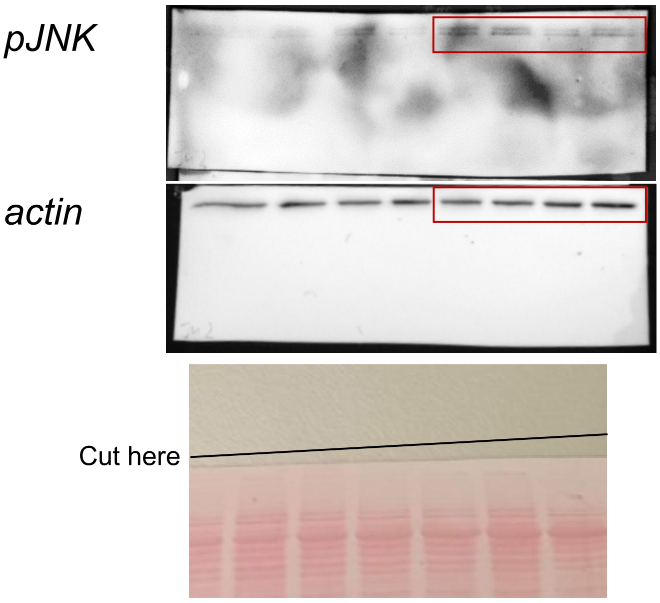

Cut here

Blots shown are lower parts of the membrane.

Original, uncropped blots of Fig.2I

p-p38

actin

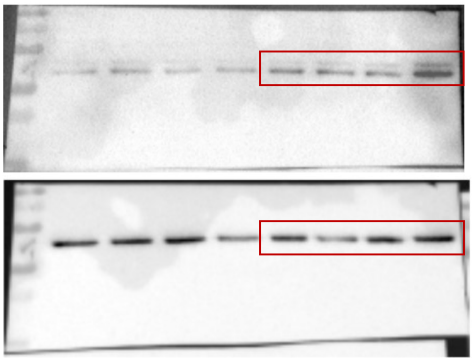

Original, uncropped blots of SFig.2H

pJNK

actin

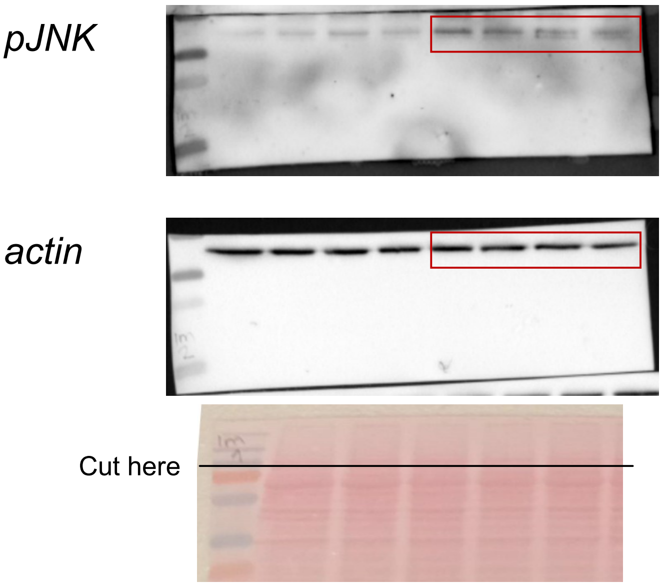

Cut here

Blots shown are lower parts of the membrane.

Original, uncropped blots of SFig.2I

p-p38

actin

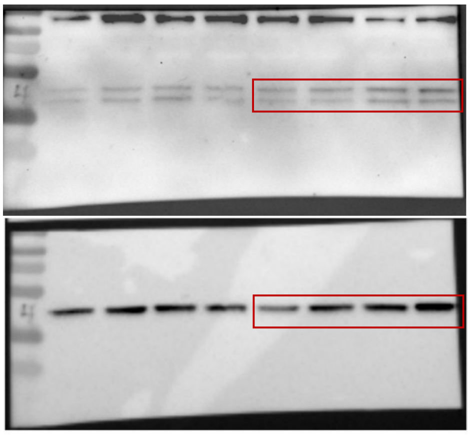

Supplement: Supplementary file 1 [file cancers-13-00511-s001.pdf]
